# Early multidisciplinary assessment was associated with longer periods of sick leave: A randomized controlled trial in a primary health care centre

**DOI:** 10.3109/02813432.2013.811943

**Published:** 2013-09

**Authors:** Lars Carlsson, Lars Englund, Johan Hallqvist, Thorne Wallman

**Affiliations:** ^1^Uppsala University, Department of Public Health and Caring Sciences, Family Medicine and Preventive Medicine Section, Uppsala, Sweden; ^2^Centre for Clinical Research Dalarna, Uppsala University, Falun, Sweden; ^3^Centre for Clinical Research Sörmland, Uppsala University, Eskilstuna, Sweden

**Keywords:** General practice, GP, multidisciplinary, primary health care, randomized controlled trial, rehabilitation, sick leave, sickness certification, Sweden

## Abstract

**Objective:**

To study the effects on sick leave from an early multidisciplinary assessment at a primary health care centre.

**Design:**

Randomized controlled trial.

**Setting:**

Patients who saw GPs at a primary health care centre in mid-Sweden and asked for a sickness certificate for psychiatric or musculoskeletal diagnoses were invited to participate. Patients included were sick-listed for less than four weeks; 33 patients were randomized either to an assessment within a week by a physiotherapist, a psychotherapist, and an occupational therapist or to “standard care”. The therapists used methods and tools they normally use in their clinical work.

**Main outcome measure:**

Proportion of patients still sick-listed three months after randomization, total and net days on sick leave, and proportion who were on part-time sick leave.

**Results:**

At follow-up after three months, in contrast to the pre-trial hypothesis, there was a trend toward a higher proportion of patients still sick-listed in the intervention group (7/18) as compared with the control group (3/15). The intervention group also had significantly longer sick-listing periods (mean 58 days) than the control group (mean 36 days) (p = 0.038). The proportion of patients who were part time sick-listed was significantly higher in the intervention group (10/18) than in the control group (2/15) (p = 0.027).

**Conclusions:**

In this study an early multidisciplinary assessment was associated with longer periods on sick leave and more individuals on part-time sick leave.

Multimodal/multidisciplinary rehabilitation has been regarded as “a gold standard” for shortening long periods of sick leave but the effect on sick leave has been questioned.

- In this RCT, early multidisciplinary assessment was associated with an increased total number of days on sick leave during the first three months.
- Early multidisciplinary assessment was correlated with a higher proportion of part-time sick listing.
- Further studies are needed to understand which patients can benefit from multidisciplinary assessment, the initiation point in the sick leave period, and what the content of the intervention should be.

## Introduction

In Sweden sick-leave issues have had high priority within the medical and political debate in recent years due to a rapid and large increase in the total number of granted sick-leave days in Sweden. In the period 1998–2003 the total number of sick-leave days more than doubled and the sum of sick- leave days and disability pension days divided by the number of persons insured in the national social insurance system increased from 33.6 days in 1998 to 43.2 days in 2003. The corresponding figures are now decreasing rapidly and in September 2011 the figure was 28.2 days. Approximately 20% of this figure was sick-leave days and 80% was disability pension days [1].

Disability pensioners report decreased quality of life and show increased mortality in several studies [2–4]. In Sweden the dominant causes of disability pension 2008 were psychiatric diseases (41%) and musculoskeletal disorders (25%). Most people in the general population who suffer from musculoskeletal pain are not sick-listed. In a study from the Swedish county of Dalarna of people who were not sick-listed, 49% of the men and 59% of the women reported “frequent pain in arm, back, or legs” [5]. The scientific knowledge of why some people are on sick-leave and others are not is scarce. Differences of opinion on sick-leave among patients, physicians, employers, and Social Insurance Agency staff have been demonstrated and may provide one explanation [6–9]. Even so doctors seldom refuse to sick-list their patients [10]. Symptom intensity and workplace requirements as well as individual differences in how symptoms affect perceived ability to work are other possible explanations.

Multimodal rehabilitation is the gold standard method in Sweden to shorten long periods of sickness certification and is included in many recommendations from health authorities [11,12]. By definition, the term “multimodal” means that professionals from more than two health care disciplines are involved, working in an integrated team [13]. The authorities have taken initiatives and invested large sums of money (two billion SKr/year 2009–2012) in accelerating and improving the rehabilitation of sick-listed individuals [12,14,15].

When it was difficult to rehabilitate patients, GPs often said that this was because rehabilitation started too late. There are no studies showing positive effects of early interventions on return to work [16]. In a report “Sjukskrivning – orsaker, konsekvenser och praxis” the Swedish Council on Health Technology Assessment (SBU) identified a need for randomized intervention studies to investigate effects of interventions on sick-leave [17]. The aim of this study was to see if GPs, with support from an early multidisciplinary assessment carried out in a primary health care setting, could help patients to achieve faster and more appropriate rehabilitation to lower the risk of long term sick-leave.

## Material and methods

The study took place at a county council-operated primary health care centre in mid Sweden. The health centre had a catchment area of 8500 inhabitants and had 4.5 full-time physician posts, one of which was vacant. One physiotherapist, one psychotherapist, and one occupational therapist made all assessments. All intervention patients met all three professionals.

Patients eligible to participate in the study were sick-listed, either full-time or part-time, according to ICD 10-diagnoses chapter V F00-F99 (psychiatric diseases) or Chapter XIII M00-M99 (musculoskeletal diseases), and had an ongoing sick-leave period of a maximum of 28 days at randomization. The inclusion process took place from spring 2007 until winter 2008/2009. The GPs invited the patients to participate in the study after the sickness certification was issued, and gave them vocal and written information. Randomization was done by the sick-listing GP by opening randomly mixed closed envelopes. Patients randomized to intervention were given an appointment within a week to meet the assessors. Controls received “treatment as usual”, which did not include this kind of early assessment. The physiotherapist performed a clinical examination of the musculoskeletal system. The psychotherapist made an assessment of the psychosocial situation at work and at home. The occupational therapist performed an assessment of the patient's general working capacity. All three therapists used the methods and tools they normally use in their clinical work (Appendix 1). For each patient, only methods judged relevant were used. The intervention did not include any treatment, but if a patient was judged to have potential to benefit from treatment, he or she was referred by the GP to standard healthcare resources.

All information from the assessments was documented in the electronic patient record and usually also discussed with the doctor who had issued the medical certificate within a week. The data on duration and extent of the sick-listing periods in the study were taken from the electronic patient records and from the records of the Social Insurance Agency. Gross and net days were calculated. All patients who were included after randomization and who did not actively decline to attend were analysed (n = 33). We called this an analysis according to “intention to treat”.

Power calculation assumed that 30% of patients sick-listed after 14 days would still be on sick leave after three months. The aim of this study was to halve the number of patients still sick-listed at three months. With a p-value of 0.05 and a desired power of 0.8, 64 subjects were needed.

Statistical analyses were performed in PASW 18 (SPSS). As the material was relatively small and not normally distributed, the tests used were non-parametric (Mann–Whitney U-test and Fisher's Exact Test). All analyses were calculated using two-sided tests.

## Results

**Figure 1.**
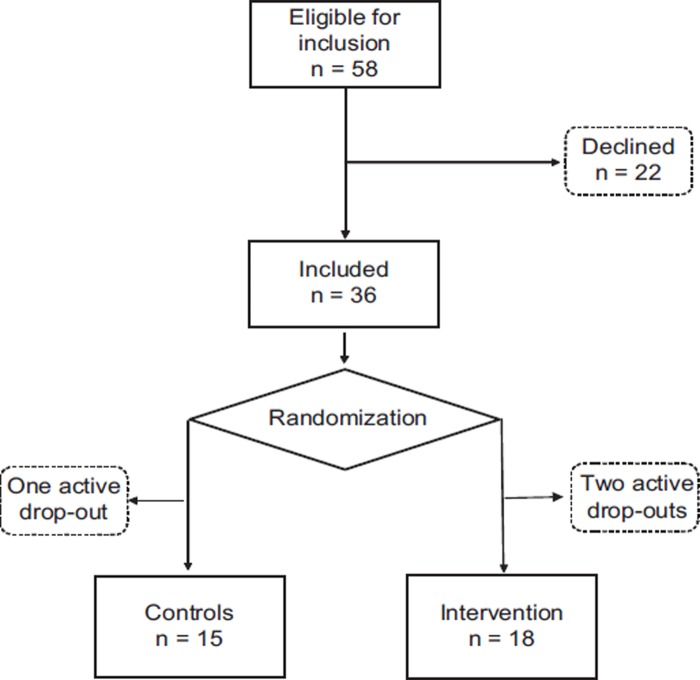
Flow chart of eligible patients invited to participate in the study and randomized participants.

A total of 58 patients were invited to take part in the study. Eight GPs recruited the patients. In all, 36 patients agreed to participate in the study and were randomized, but three women (one in the control group and two in the intervention group) later withdrew from participation before assessment; 33 patients were finally committed to the study (Figure 1).

The randomization resulted in groups that were similar regarding age, sex, and diagnoses on the sickness certificate (Table I). In the intervention group at randomization 15 of 18 were on full-time sick-leave and in the control group 14 of 15. Two in the intervention group and one in the control group were unemployed. Among patients who declined participation (n = 22) before randomization no significant difference in age compared with the study participants was found. In the analysis, seven out of 18 in the intervention group were still sick-listed at three months and three out of 15 in the control group (Table II). The number of days on sick-leave was significantly higher (p = 0.038) in the intervention group, with a mean value of 58 days, as compared with 36 days in the control group. The difference was slightly smaller and not significant (p = 0.070) when net days of sick-leave during the three first months were analysed. Net days of sick-leave were calculated as number of days in the period multiplied by the percentage of sickness certification. This can be explained by the fact that the proportion of individuals who were sick-listed part-time for a period during these three first months was significantly higher (p = 0.027) in the intervention group, 10 out of 18, as compared with two out of 15 in the control group.

**Table I.** Comparison of age, sex, and diagnosis on sick note between all invited patients and those who participated in the study.

	n	Age	Women%	Pain	Psych.	Pain+ Psych.
Invited to participate	58	46	72	*	*	*
Declined participation	22	46	82	*	*	*
Randomized	36	46	67	27	6	3
Control group	15	48	67	11	3	1
Intervention (intention to treat)	18	44	61	13	3	2
Intervention (completed)	16	45	62	11	3	2

**Table II.** Sick-leave measures at three and 12 months for intervention and control groups.

	Intervention (n = 18)	Control (n = 15)	
Still on sick leave after three months	7/18	3/15	p = 0.283
Total number of gross sick leave days in the first three months			
Mean (SD)	58 (32)	36 (33)	p = 0.038
Median	65	21	
IQR	69	51	
Range	81	87	
Total number of net sick leave days in the first three months			
Mean (SD)	48 (32)	32 (29)	p = 0.070
Median	42	21	
IQR	73	39	
Range	84	87	
Number of individuals who were on partial sick leave 0–3 months	10/18	2/15	p = 0.027
Still on sick leave after 12 months	4/18	1/15	p = 0.346
Total number of gross sick leave days 3–12 months, mean (SD)	91 (123)	58 (95)	p = 0.727
Total number of net sick leave days 3–12 months, mean (SD)	77 (109)	37 (62)	p = 0.580

Notes: (SD = standard deviation, IQR = Interquartile range).

## Discussion

In contrast to our hypothesis, early multidisciplinary assessment was found to significantly increased days on sick-leave in the first three months. The proportion of people on part-time sick-leave was significantly higher in the intervention group, but sick-leave was longer in this group even when counted as net days.

The strength of this study is the randomized design and the fact that it was carried out in a primary health care setting, where many sickness certification periods begin. The information on sick-leave days was complete, as both data from the electronic patient records and data from the Social Insurance Agency were included.

Weaknesses are that data on sickness absence before inclusion are missing but randomization probably minimized any differences. The fact that this study could not randomize the planned number of individuals and that only one centre was involved adds further weakness. The relatively large number of patients who declined participation before randomization could be explained by the fact that some patients were on sick-leave for uncomplicated ailments with a good prognosis and considered the extensive assessment unnecessary. Another possible explanation could be the media debate on high sickness absence, which was very intensive when the study was being conducted. This may have been a reason for some patients eligible for inclusion to abstain, because they were concerned that an expanded assessment would question their need for sickness absence. This weakens the generalizability of this study. On the other hand the study gains generalizability from having been performed in an average Swedish, county council operated, primary health care centre as regards size, population, and access to doctors and other rehabilitation resources.

Some studies carried out at pain or rehabilitation clinics have shown effects of multidisciplinary/multimodal treatment later in the sick-leave period (after four weeks) on patients sick-listed for low back pain [18]. The results from our study, indicating that early multidisciplinary assessment had the opposite effect, are not unique. In a Swedish study of an early multidisciplinary rehabilitation programme for neck and shoulder disorders lasting eight weeks, significantly more days of sick-leave in the first year were noted [16]. “Raskijobb” was a Danish study aiming to prevent sickness absenteeism but the effect was an increase of 15% more sick-leave days [19]. In a Swedish randomized evaluation of a “special resource team” for patients at risk of prolonged sick-leave, GPs, physiotherapists, behavioural therapists, and officials from the Social Insurance Agency made assessments of rehabilitation needs. The effect was a 20% increase in the number of sick-leave days as compared with a control group [20]. “SASSAM” is a method often used at the Social Insurance Agency offices in Sweden to support the patient's return to work and to assess the need for rehabilitation efforts. This method does not include any treatment. In a randomized study on early “SASSAM” there was a non-significant tendency towards longer sick-leave periods with this intervention [21]. In a Dutch study, efforts to reduce sickness absence also resulted in increased sick-leave days [22].

Two evaluations of “Rehabgarantin”, where the Swedish government invested almost 1 billion SKr/year from 2009 to 2012 with the most common measure being multimodal rehabilitation for musculoskeletal pain, showed no effect on total sick-leave [23], or increased sick-leave days [24].

There are several possible explanations for the result of this study. Hanne Hollnagel, a Danish professor of Family Medicine, stresses the importance of focusing on the patient's own resources and opportunities in the consultation instead of only on symptoms and problems [25]. The extensive assessment by the physiotherapist, psychotherapist, and occupational therapist early in the sick-leave period might have the effect of focusing more on symptoms and problems. This could adversely affect “recovery expectations”, “internal locus of control”, “fear-avoidance”, “catastrophizing”, “self-perceived poor health”, and “self-efficacy”, all factors shown to be predictors of return to work [26–29].

Part-time sick-leave has been shown to have a potential to be a way back to work as well as posing a risk for extending the sick-leave [10,30]. In this study 3/18 in the intervention group and 1/15 in the control group were sick-listed part time at inclusion. Correcting for this difference, by excluding part-time sick-listed individuals at inclusion, did decrease the p-value for the difference in total days of sick-leave between the two groups. This is an argument against the idea that the difference in the proportion of people on part-time sick leave at inclusion could explain the results. Another possible explanation could be that the information that emerged at the multidisciplinary assessment process was not properly handled in the subsequent rehabilitation and sickness certification process that took place outside the study within ordinary healthcare resources.

## Conclusion

In this study, the total number of sick-leave days was significantly higher in the intervention group with early multidisciplinary assessment. Further randomized studies are needed to obtain better knowledge of which patients can benefit from multidisciplinary assessment in primary health care, when in the sick-leave period this should optimally be performed, and what the content of the intervention should be.

## Ethical approval

The study was approved by the ethics committee at Uppsala University registration number 2006/305.

## Appendix 1. Methods and tools available for use in assessment when found appropriate

Physiotherapist:

1. Steven Linton “Orebro musculoskeletal pain questionnaire” (OMPQ) [34].
2. Painful template and VAS scale.
3. “Waddel signs” of psychosomatic pain.
4. McKenzie forms of cervical and lumbar spine examination.
5. Axelina form for shoulder status.
6. Physical work capacity.
7. FIQ forms for grading pain, fatigue, anxiety, sleep etc.

Psychotherapist:

1. The form “Living history and current problems”.
2. The form “SCL-90” rating scale of various disorders.
3. In the case of suspected abuse the questionnaire “AUDIT”.

Occupational therapist:

1. “WRI-S” to identify psychosocial and environmental factors that discourage return to work.
2. “WEIS-S” which provides a picture of how the person perceives his/her psychosocial and physical work environment (for people in work).
3. “My view”, a self-report instrument, focusing on task ability, physical and social environment, values and priorities (for people out of work).
4. “OCAIRS-S” which provides a picture of the person's life situation.
5. Cognitive assessment if judged necessary, with the instrument RBMT (the Rivermead behavioral tests), Trail Making Test A/B, AQT, Social sequences (sequential organization), clock test, MMT.
